# The Impact of Managerial Changes on Physical Performance in Elite Soccer Players

**DOI:** 10.3390/sports13070213

**Published:** 2025-06-30

**Authors:** Dennis Petrov, Koulla Parpa, Marcos Michaelides

**Affiliations:** Sport and Exercise Science, UCLan University of Cyprus, Pyla 7080, Cyprus; dpetrov1@uclan.ac.uk (D.P.); mmichaelides@uclan.ac.uk (M.M.)

**Keywords:** managerial changes, physical performance, coaching regimens

## Abstract

This study aimed to examine whether managerial changes and their training methodology influence the physical attributes of soccer players and determine if these changes significantly impact the overall physical performance of the team. Twenty-seven male elite-level football players competing in the Eastern Mediterranean region (age: 28.12 ± 5.5 years, height: 179.3 ± 6.25 cm, body mass: 75.8 ± 6.6 kg) participated in this study. To analyze the impact of managerial changes on elite football players’ physical performance, this study evaluated and compared physical attributes during weekly microcycles and official games across three different coaching regimes over an entire season. Data were collected using a 10 Hz GPS tracking technology and included the following external load (EL) parameters: total distance, high metabolic load distance, high-speed running, sprint distance, accelerations, and decelerations. A one-way Analysis of Variance (ANOVA) was utilized to assess differences in physical performance across the three coaching methods. Significant differences were evident in high metabolic load distance during games [*F*(2,27) = 7.59, *p* < 0.05]. High-speed running distance also varied significantly across the three coaching regimes, both during training sessions [*F*(2,27) = 5.89, *p* < 0.05] and games [*F*(2,27) = 4.31, *p* < 0.05]. Furthermore, sprint distance showed significant differences during training [*F*(2,27) = 4.62, *p* < 0.05] and games [*F*(2,27) = 3.37, *p* < 0.05]. The findings of this study suggest that managerial changes can have a significant effect on the physical performance of soccer players. The results highlight the importance of aligning coaching strategies with physical conditioning techniques for optimizing performance. These findings provide a deeper understanding of the potential benefits and risks associated with managerial changes in professional soccer. Nevertheless, a limitation in this study is that all metrics of EL were interpreted as absolute values rather than relative-based threshold values, which may affect the interpretation of the players’ physical capacities.

## 1. Introduction

Soccer is a highly popular sport, known for its combination of tactical intelligence, ball skills and athleticism, which attracts significant attention from coaches and researchers [1]. During the last decade, there has been a significant increase in the technical and tactical demands of the game, particularly emphasizing the physical conditioning of professional soccer players [2,3]. Nevertheless, increases in physical demand also present a considerable risk of injury, mainly in the lower limbs [4]. Soccer injuries in the lower limbs account for a significant proportion of total soccer-related injuries, with a percentage occurrence of 37% in the hamstrings, 25% in the ankle, and 17% in the knee [5,6]. As a result, soccer places increasing physical and technical demands on the players, especially depending on their position, to help teams perform well and maintain individual efficiency throughout the games [7]. Moreover, superior physical performance in soccer competitions is closely related to the physical stimuli applied during training sessions [8]. The demanding nature of these requirements and the constant goal of improvement place considerable pressure on soccer players to consistently perform and adapt. This highlights the crucial role of coaches and managerial staff in guiding players and supporting their development [9].

As a result, trainers and coaches are consistently implementing various training models with specific training techniques to enhance performance in official games. Consecutively, the managerial staff upholds a key and pivotal role in constructing a team’s strategic identity and approach to the game [10]. Different managers implement specific and unique methods in training sessions and official games, striving to constantly elevate the team’s overall performance. Nevertheless, high performance in soccer is measured by success in competitions and the number of games won. Due to this great demand for success at the elite level, managers and coaching staff are constantly required to maintain or elevate their team’s performance, success rate, and league ranking. Poor results during competition can often lead to a sudden change in managerial staff at any phase of the competitive season (e.g., pre-season, mid-season, play-offs). Consequently, recent data indicate that the managerial transition in professional soccer is high [11]. In a recent study on managerial transitions in the Brazilian Soccer League, it was evident that the median appointment for a manager was approximately 16 games [12]. An additional study reported that dismissals of soccer managers are more likely to occur during mid-season periods [13]. In addition, a study on managerial dismissals in the “Big Five” European soccer leagues recorded 55 coaching changes only during the 2017/18 competitive season [11]. A change in the coaching staff and the arrival of a new manager are frequently expected to rectify the strategic model of playing style with innovative player organization and development [14]. However, a new manager can also negatively influence a team’s physical performance and their health-related outcomes [15]. For instance, if the new manager significantly increases or decreases the physical load during training, this can result in a risk of overtraining or undertraining, and therefore, increase the risk of injury [16]. Training sessions have a major effect on the overall performance and the health-related outcomes in official matches. Moreover, as the head coach plays a pivotal role in the planning and design of the technical, tactical, physical, and psychological aspects of a soccer team, they hold significant responsibility for managing optimal team performance and avoiding injuries.

To date, several studies have highlighted the various outcomes and effects that a coaching change can have on a team [17]. While some researchers found positive effects from managerial changes in performance [11], others have failed to identify similar success following the appointment of a new manager [18]. Nevertheless, these studies focused primarily on the success rate in official games, measuring psychological and behavioral parameters, and categorized teams’ performance based on their wins/losses and ranking. While substantial research has been devoted to studying the technical and tactical components of the game, there is a distinguished gap in the understanding of how different coaching methods and managerial staff variations affect the physical attributes and overall performance of soccer players [19]. The interpretation of a physical training model and player development can vary widely between coaches depending on their primary focus and game strategy [20]. The players’ physical output is a fundamental component of their performance capabilities. Any changes in managerial organization can have a direct or indirect effect on their performance and development [21]. Consequently, a change in the managerial staff can result in both a psychological and a physiological shift in the overall methodological application and function between coaches and players. Therefore, such changes are of great importance, as they can significantly influence a team’s physical and psychological identity.

It is important to note that a managerial transition is not considered a simply organizational event but also a significant occupational stressor [22]. Occupational stressors can have a profound effect on mental health, cognitive clarity, and professional awareness [23]. It is imperative to highlight that occupational stressors due to such transitions can increase anxiety, nervousness, and decrease the overall chemistry and well-being between players and coaches, resulting in a detrimental factor for the team’s overall success [22].

Therefore, this study aimed to examine whether managerial changes influence the physical attributes of soccer players and determine if these changes significantly impact the overall physical performance of the team. We hypothesized that managerial changes would affect the players’ EL and that these alterations would yield significant implications on the collective physical performance of the team, as presented in previous research [24].

## 2. Materials and Methods

### 2.1. Experimental Approach to the Problem

To analyze the impact that managerial changes impose on the physical performance metrics of elite football players, the current study was designed to evaluate how physical performance metrics varied across three distinct coaching methodologies implemented during a full season. The current study aimed to assess potential shifts in team dynamics and physical performance between the players that could potentially result from the managerial changes. This experimental setup involved 27 male elite-level football players competing in a professional league, therefore ensuring that the subjects were representative of a population which would typically be affected by managerial changes. The subjects were consistently monitored during the weekly microcycles and official games of the season to obtain a comprehensive performance profile for each managerial staff. Performance metrics were recorded using 10 Hz GPS tracking technology for all subjects under the 3 different coaching staff, to accurately assess the external load (EL) for each player. Data from the EL was collected from all training sessions and competitive games over the complete length of the season. Each of the 3 different managerial regimes was assessed and compared in terms of coaching strategy, training procedures and physical conditioning methods. Total distance, high metabolic load distance, high-speed running, sprint distance, accelerations, and decelerations were the central parameters recorded for the aims of this study.

### 2.2. Participants

Twenty-seven male elite-level football players from Division 1 (age: 28.12 ± 5.5 years, height: 179.3 ± 6.25 cm, body mass: 75.8 ± 6.6 kg) participated in this study. The study examined their physical performance during the weekly microcycles and official games throughout an entire football season. Furthermore, data were collected only from outfield players (excluding goalkeepers) who participated in >90 min of total duration in official games. The sample for the current project consisted of 36 competition recordings and 169 recordings of training sessions from up to 5 days before matchday 5 (MD − 5) (i.e., MD − 5, MD − 4, MD − 3, MD − 2, and MD − 1). Training sessions were conducted during the morning hours between 09:00 and 11:00, and the official games took place in the evening hours between 18:00 and 21:00. The study was approved by the University of Central Lancashire Science, Technology, Engineering, Medicine and Health (STEMH) ethics committee board and the Cyprus National Committee on Bioethics (CNCB). Furthermore, this study adhered to ethical guidelines based on internationally recognized standards.

### 2.3. Procedures

Recordings of external load parameters were analyzed using 10 Hz GPS tracking software on internal devices (WIMUPRO, RealTrack Systems, Almeria, Spain). The efficiency and reliability of these devices have been successfully evaluated in recent research [25]. Detailed intercession of separating drills and acquiring recordings of active time was executed in real-time (SVIVO software, version 2021.211.2.0, RealTrack Systems, Almeria, Spain). Following the conclusion of each session, all data and activity markings were transferred to a computer and analyzed by SPRO (Version 989, RealTrack Systems, Almeria, Spain).

### 2.4. Coaching Profiles

The team played 14 official games (6 home and 8 away) and completed 58 training sessions under the first coaching staff; 8 games (3 home and 5 away) and 46 training sessions under the second; and 14 games (7 home and 7 away) with 65 training sessions under the third coaching staff. All 3 coaches implemented the “1-4-3-3” playing formation system during the official games. However, each coaching staff presented a different method of training protocol with specific exercise techniques, drill progressions, and their execution on various pitch dimensions. The first coaching staff (C1) had organized the training sessions within an average range of 68.3 min per training session. The detailed training modalities and structure of each training day for C1 are presented in Table 1. The C1 maintained a consistent first half of every training session, lasting ≈35% of the entire session with a routine warm-up protocol, which consisted of movement activation exercises, dynamic stretches, and a short-spaced passing drill (pitch sizes: 9 × 14 m and 14 × 18 m). There were no additional collective running drills, individual speed drills and/or strength-related specific exercises during the first half of the training session. The C1 highly relied on implementing small-sided games (SSG) (pitch sizes: 30 × 25 m and 46 × 40 m), which accounted for ≈25% of the sessions’ duration on MD − 3, MD − 2 and MD − 1. On MD − 4, the C1 primarily implemented prolonged SSGs, lasting for up to 50% of the session’s duration. Furthermore, the C1 devoted a large proportion of training time (≈40%) to tactical play on MD − 3, MD – 2, and MD − 1. Individual shooting and scoring drills were only applied on MD − 1; however, their frequency was inconsistent.

The second coaching staff (C2) had organized the sessions within an average range of 63.8 min per training. The detailed training modalities and structure of each training day for C2 are presented in Table 2. The C2 relied on a warm-up routine contextual to the objective of the specific training (i.e., short-spaced exercises including the ball for strength and agility-related sessions and long-spaced exercises frequently excluding the ball for speed and endurance-related sessions), lasting ≈30% of the duration of sessions MD − 2 and MD − 1. The first half of training sessions MD − 4 and MD − 3 were heavily devoted to fitness-based activities on the pitch (running drills, resistance exercises, and agility drills), which accounted for ≈50% of the total training duration of the specific sessions. Furthermore, C2 relied on a thorough training design containing SSG (pitch sizes: 46 × 46 m; 50 × 44 m and 60 × 50 m) in all training sessions, lasting ≈40% of the trainings’ duration, and applied tactical play scenarios only during MD − 2 and MD − 1 lasting ≈10% of the sessions’ duration.

Finally, the third coaching staff (C3) organized the training sessions within an average of 60 min per session. The detailed training modalities and structure of each training day for C3 are presented in Table 3. The C3 had a pre-training routine in the form of 10 min presentations on the content for the training design, drill requirements, and tactical formations. The first part of every training consisted of a routine warm-up protocol for muscle activation exercises and dynamic stretches lasting ≈10% of the session’s duration. Nevertheless, C3 had spent little to no time on fitness activities (i.e., drills without the ball) and had only devoted time to passing and scoring drills on MD − 3, which generally accounted for ≈25% of the session’s duration. Furthermore, C3 had strictly developed the pieces of training to imitate game-play situations, resulting in the design of large-sided games (LSG), (pitch sizes: 80 × 70 m and 100 × 74 m) throughout every training, lasting ≈60% of the sessions’ duration. Finally, the C3 implemented tactical play during MD− 2 and MD − 1, lasting ≈10% of the sessions’ duration.

### 2.5. Analyzed Parameters

The total training and game duration were recorded in minutes, the total distance (TD) in meters and the high metabolic load distance (HMLD), that is, the distance covered when the metabolic power was over 25.5 W/kg, also in meters [7]. Furthermore, the HMLD value corresponds to the moments when running is intense (>5.5 m/s^2^), including high acceleration activities. Additionally, measures included absolute high-speed running (HSR), which represented the distance in meters covered at greater than 21 km/h and the sprint distance in meters, which was recorded when the running speeds were greater than 24 km/h. Finally, measurements also included the number of accelerations (ACCc) and decelerations (DECc) and the respective distances covered during accelerations (ACCd) and decelerations (DECd).

### 2.6. Statistical Analyses

The statistical analysis was performed using the SPSS Statistical Package (Version 26.0 for Windows; SPSS Inc., Chicago, IL, USA). Descriptive statistics were presented as means and standard deviations for the physical parameters among the 3 coaching staff during training sessions and games. The study quantitatively assessed the effects of 3 different coaching methods on various physical performance metrics in elite soccer players using One-way Analysis of Variance (ANOVA), followed by post hoc Bonferroni adjustment for multiple comparisons where applicable. The level of significance was set at *p* ≤ 0.05.

## 3. Results

The study quantitatively assessed the effects of three different coaching methods on various physical performance metrics in elite soccer players. Descriptive characteristics for the players under each of the three coaching teams are presented in Table 4.

### 3.1. Duration

The one-way ANOVA test revealed no significant differences between the three coaching regimes, *F*(2,27) = 0.50, *p* > 0.05, demonstrating a consistent training duration across the analyzed sessions. However, C3 highlighted a notably lower duration in training sessions of 9.6% compared to C1. The match duration was not analyzed due to the official and standard duration of each competition.

### 3.2. Total Distance

The TD covered by the players during training sessions and games was consistent and did not present any significant differences between the three coaching protocols [*F*(2,27) = 0.11, *p* > 0.05]. The results for TD and the absence of statistically significant differences could be due to the decreased sensitivity to tactical and technical-based differences between coaching regimens, as TD is a measure of activity volume and not intensity.

### 3.3. High Metabolic Load Distance

Significant differences were evident in HMLD for MD [*F*(2,27) = 7.59, *p* < 0.05], underlining a distinct higher intensity performance in this metric for the regime of C3. No significant differences were observed between the three coaches for the same metric during training sessions.

### 3.4. Accelerations and Decelerations Count and Distance

No significant differences were observed for ACCc/DECc and ACCd/DECd between the three coaches for both training sessions and games. There were no significant differences for ACCc during the games between C1 and C2 [*F*(2,27) = 3.48, *p* = 0.063]. Also, the results obtained from DECc further exhibited no significant differences between C1 and C2 [*F*(2,27) = 3.50, *p* = 0.062].

### 3.5. High-Speed Running and Sprint Distance

**Figure 1:**
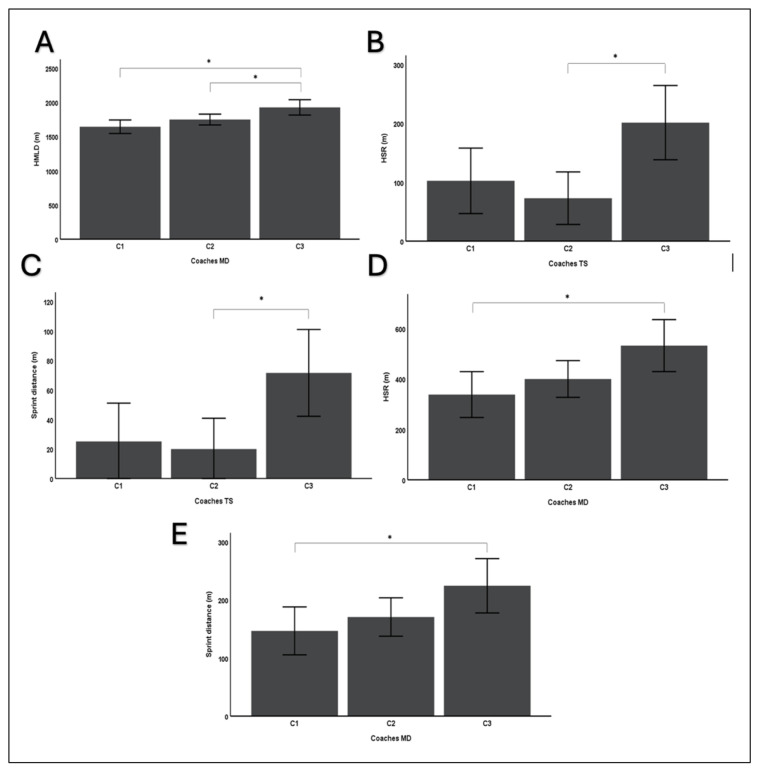
Data are presented across (**A**) Game estimations performed for HMLD (the distance covered in meters by a player when the metabolic power is over 25.5 W/kg); (**B**) Training sessions’ estimations performed for HSR (distance covered > 21 km/h);(**C**) Training sessions’ estimations performed for Sprint distance (distance covered > 24 km/h); (**D**) Game estimations performed for HSR (distance covered > 21 km/h); (**E**) Game estimations performed for Sprint distance (distance covered > 24 km/h); * (*p* < 0.05).

The statistical analyses demonstrated a significant difference in HSR during the training sessions between the three coaches [*F*(2,27) = 5.89, *p* < 0.05]. Specifically, the results for C3 were significantly higher for this metric than for C2. Furthermore, no significant differences were evident for the same metric, as observed between C3 and C1 [*F*(2,27) = 5.89, *p* = 0.069]. Similar results were observed for sprint distance during training sessions [*F*(2,27) = 4.62, *p* < 0.05], classifying C3 with significantly higher sprint distance during training sessions than C1 and C2. These findings between the three coaches were replicated for HSR during games [*F*(2,27) = 4.31, *p* < 0.05], classifying C3 with significantly higher HSR performance compared to C2, and no significant differences compared to C1 (*p* = 0.066). Lastly, the results demonstrated that C3 recorded significantly higher sprint distance [*F*(2,27) = 3.37, *p* < 0.05] during games compared to C1 (*p* = 0.05). Significant differences for games and training sessions between the three coaches are presented in Figure 1.

**Figure 2:**
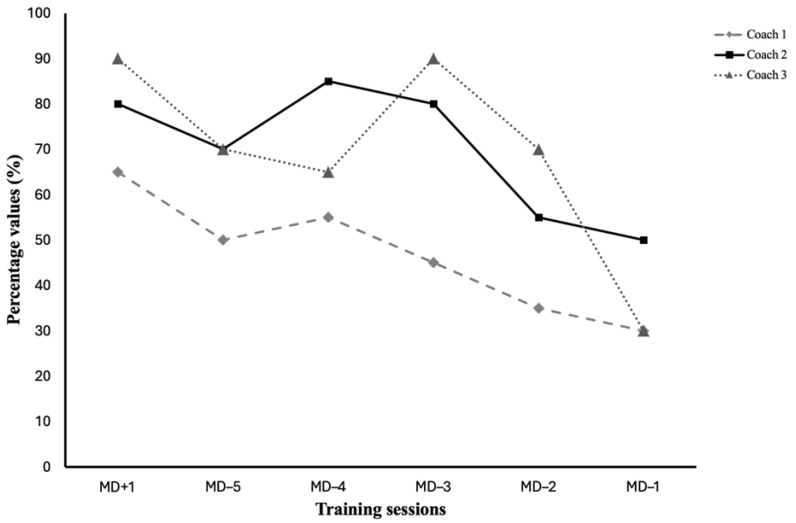
Weekly microcycles’ average external load in different training sessions relative to the average EL of matches of the three different management teams. Values for all three management teams are presented as approximate percentage values.

The average EL in the different training sessions compared to the average EL of games for each coaching staff is presented in Figure 2.

It is important to mention that all metrics of EL were interpreted as absolute values rather than relative-based threshold values. This limitation may reduce the sensitivity of detecting true-player inter-individual differences and their physical output across the different coaching regimes.

## 4. Discussion

This study investigated the physical performance of elite soccer players’ competitive season under the training management of three different coaching staff during a complete competitive season. Following the conclusion of the competitive season, each coaching staff member was categorized according to their wins (W), losses (L), and draws (D). The C1 contributed to (5 W, 6 L, 3 D), C2 recorded (3 W, 3 L, 2 D), and C3 added (8 W, 2 L, 4 D). The interplay of winning and losing in soccer performance is often the main reason for managerial changes, as organizations tend to induce a “shock effect” principle, where a new coach is expected to embolden the players’ focus and motivation, provoking positive dynamics and wholesome chemistry within the team [26]. The conclusion of the season resulted in the evident outcome that C3 contributed the most efficient record in terms of winning percentage, at approximately 57%. A recent longitudinal study suggested that the “shock effect” does indeed enhance the winning percentage and significantly improves the short-term performance of soccer players for up to 10 games post-coaching change [11]. However, the authors only assessed the differences in points awarded from match outcomes, team budget, and the coaches’ professional backgrounds (i.e., former player or not). The authors did not collect any physical data from the games and training sessions.

To our knowledge, this is the first study to quantitatively analyze and compare the physical metrics between elite soccer players under three different coaches within a complete competitive season. Each of the three different coaches held a distinctive technique in the design of their weekly microcycles (Table 1, Table 2, Table 3 and Table 4, Figure 2). The results from the current study presented no significant differences in TD, ACC/DEC count, and distance during training and games between the three coaches. The reasoning behind this could have been due to ACC/DEC being less influenced by tactical differences, such as game formations and player positioning on the field. Such actions are more likely to be affected by technical, game-specific actions such as transitions, defensive and offensive pressing, and duels. Furthermore, a limiting factor could have been the relatively small sample size, which may have reduced the statistical power of detecting significant differences in such highly variable metrics as ACC/DEC. Nevertheless, C1 recorded substantially greater ACCc/DECc during official games compared to C2 (*p* = 0.06), suggesting a more dynamic approach under the C1 regimen. In contrast, C1 recorded the lowest values for HMLD, HSR, and SD in games, indicating a lower locomotor performance. Recent research explained that overtraining from explosive parameters such as ACC/DEC could result in an insufficient stimulus and undertraining for locomotor variables such as HSR and SD, as demonstrated in the current study [27]. Furthermore, the TL accumulated from ACC/DEC during training sessions can negatively influence the overall performance of players in official games, as it may lead to fatigue and injury [28]. In the current study, the average distance covered in ACC/DEC from all three coaches revealed a 47% greater ACCd and a 66% greater DECd in games compared to training sessions. Our results revealed an approximate ratio of ≈1:2 between training sessions and games for ACCd/DECd, and a ratio of ≈1:3.5 for ACCc/DECc. These findings replicate recent research, demonstrating a ratio of ≈1:4 for TD and ACC as arbitrary units (AU) between training sessions and games [3,28]. Further results from the current study established a ratio of ≈1.3:1 for HSR, ≈0.8:1 for SD, and ≈1:1.2 for HMLD between training sessions and games. Recent studies address these findings by reporting variations for HSR between 0.2 AU and 2.3 AU, and ratios for SD between 0.03 AU and 1.3 AU [3,29]. This outcome could explain the rationale behind the reduced values in HMLD, HSR, and SD for C1 and why C2 and C3 presented opposite results.

After assessing the locomotor and metabolic performance from the three coaches, the results indicated that C3 had significantly higher values in HMLD, HSR, and SD, in games compared to C1. According to a recent systematic review, HSR and SD were highly variable between games and significantly depended on players’ position (i.e., Midfielders, Strikers, etc.) [24]. Thus, a greater number of games played could have increased the variability between the three coaches. In the current study, however, game data were obtained from 14 official games from both C1 and C3. As a result, both groups apprehended the same set of game data; consequently, their differences occurred due to the content of their data and not its magnitude. The comparison of the training sessions between the three coaches concluded that C3 recorded significantly higher values for HSR and SD than C2, but no significant differences in the same parameters when compared with C1 (*p* = 0.07). The exercise stimulus applied for high-velocity movements during training sessions is reciprocal to the extent of high-velocity activities performed during games [30,31]. Our findings indicated that C3 recorded >50% greater results in HSR and >65% higher results in SD than C1 and C2 during training sessions. Consequently, applying a higher spur of HSR and SD in training sessions may contribute to the locomotor performance of soccer players during games. A principal component for achieving HSR and SD during training sessions is contextualized exercise drills [32]. There is a plethora of theories and research on the most precise and most effective techniques for safely increasing HSR and SD in official games without increasing the risk of fatigue and injury [8,30,33]. The complexity of soccer, however, narrows the ability to distinguish among these techniques, as HSR and SD are highest in variability (60–120%) [34] during training sessions, and (20–30%) during official games compared with other physical parameters [35,36]. Managing these variations in training sessions and increasing the accuracy in training intensity for HSR and SD are central points for fitness coaches and sports practitioners. Recent research demonstrated that a player’s physical output is more severely depressed following a peak in high-velocity movements compared to a sequence of accumulated TD and ACC/DEC [37]. An excessive decline in the physical output will require a longer recovery period and can increase fatigue levels. In other words, soccer players should be physically prepared specifically for high-velocity movements during training sessions to increase their proficiency for greater HSR and SD, and faster recovery between speed bouts. Recent research suggested that one of the most effective methods for achieving HSR and SD is to design drills which imitate the intensity and magnitude of an official game [38]. Consecutively, designing drills with broader and longer pitch measurements would increase the ability of soccer players to exceed their initial acceleration phase and extend into a higher velocity movement, thus achieving HSR and SD [27]. This theory correlates with our findings as C3 had predominantly implemented LSG (pitch sizes: 80 × 70 m and 100 × 74 m) as a fundamental training objective and was associated with the highest results for HSR and SD in official games. Recent research described that sided games in large formats (>80 × 55 m) can induce HSR and SD as they allow a player to cover a larger range of distance (i.e., 225–300 m^−2^) [38]. The authors further elaborate on the importance of psychophysiological factors (i.e., motivation, psychological drive, and competitiveness) and how they are triggered during LSGs. The literature suggests that side games can mentally stimulate players and increase their physical performance during training sessions [27]. Although some drills’ main objective is achieving HSR and SD (i.e., speed drills without the ball), players often decline to perform at their maximum. This can result in undertraining for HSR and SD as players will not reach the required stimulus designed to acquire the specific training adaptation [39].

In the current study, C3, who held structured pre-training briefings, saw the most significant improvements in HSR and sprint distance. These briefings are likely to have helped align the team’s efforts and fostered a cohesive team environment. Although psychological impacts were not measured in the current study, the improvements in physical performance metrics suggest that clear leadership and positive team dynamics played a critical role in overall performance. Effective leadership involves not only setting clear expectations but also actively engaging with the players to build trust and mutual respect. Regular team meetings, transparent decision-making processes, and open lines of communication can help to address players’ concerns and foster a sense of unity. Furthermore, recognizing and valuing each player’s contributions can enhance their commitment to the team’s goals. By creating an environment in which players feel supported and valued, managers can significantly enhance team dynamics, leading to improved performance on the field. Players’ responses to managerial changes vary significantly based on individual psychological factors such as personality, resilience, and previous experiences. Some players thrive under new management, viewing it as an opportunity to prove themselves, while others struggle with the change. Psychological resilience and the ability to cope with change are critical factors in determining how players respond to new managerial appointments [40]. As a result, it is important to note that, while these clarifications regarding leadership style and psychological factors are supported by existing literature, no psychological metrics were measured in this study.

Lastly, our findings did not reveal a significant difference in the duration of the training sessions between the three coaches. Nevertheless, it was evident that the duration of the C3 training sessions, of an average of 60 min per session, was approximately 10% shorter than that of C1, with an average of 68.3 min per session. Recent research explains the importance of training duration, as prolonged training sessions can result in an increase in psychophysiological factors and can be detrimental to the physical objective on the specific training day [21]. The authors explain that this issue typically occurs due to an extended duration of disbursed drill instructions and miscommunication during tactical formations. Moreover, this could have also affected HSR and SD during training sessions. In the current study, C3 incorporated brief pre-training presentations 10 min before each training session to introduce the theoretical training objectives and structure for each drill. This could have resulted in the reduced overall duration during the training sessions of C3.

### Limitations

This study comes with limitations. The thresholds used were interpreted as absolute values and not relative. According to a recent study, absolute thresholds fail to distinguish a player’s full physical capacities, which would, therefore, misinterpret the overall external load during training sessions and games [24]. Furthermore, the current study did not differentiate the results according to the players’ position. Recent research has shown significant differences in GPS parameters between central defenders and full-backs during games and training sessions. [41]. Furthermore, game outcomes which can essentially impact the EL in a soccer game [8] were not considered in this study. Additional contextual factors, such as game location for home and away games, and opponent strength, were also not analyzed. Research revealed significant increases in HSR and SD when facing a higher-level opponent. Additionally, past research found that soccer players recorded significantly less HSR and SD during a win and a draw in official games [14].

## 5. Conclusions

This study offers a novel quantitative comparison of physical performance parameters in elite soccer players under the management of three different coaching regimes throughout a complete competitive season. Our findings demonstrate that the coaching strategy of C3 was superior for HSR and SD metrics during official games. This highlights the specificity and efficacy of tailored training drills and exercises that replicate game intensity, particularly through the application of large-sided games, ultimately contributing to enhanced game performance. Our study also underscores the complexity of high-velocity movements and how their frequency during training sessions can have a significant impact during competition.

In addition, examining the impact of game outcomes in relation to physical load offers a promising approach to understanding the dynamics of competitive soccer performance. Our study provides valuable insight into effective training strategies in elite soccer, emphasizing the importance of coaching protocols in maximizing players’ physical capabilities and optimizing overall performance.

## 6. Practical Applications

Based on our findings, we encourage soccer practitioners to implement LGS (e.g., >80 × 70 m) in their training design to effectively stimulate HSR and SD demands. LSG imitate game-play scenarios, increasing space on the field and game realism, while allowing the players to expand their running and therefore, reach higher velocities. The findings of the current study suggest that soccer organizations should implement a structured integration of protocols for regular performance monitoring, data collection for psychological markers (i.e., stress and anxiety), and enhanced communication between staff, specifically during a managerial transition period. Performance monitoring is crucial, as increases in HSR and sprinting can increase the risk of injury, especially during acute spikes due to a higher intensity imposed by a new manager. Soccer organizations should incorporate GPS metrics with wellness markers to assess and manage the total load during training to maximize gradual adaptations. Organizations should implement individualized thresholds for key metrics such as HSR, sprint distance, and HMLD to increase the validity and reliability of the accumulated load from these metrics and maximize the coherence of their interpretation. Finally, coaches should deliver clear instructions in pre-training briefings on what would be expected in the training session. This could improve the players’ understanding during training, increase their confidence and motivation, and improve the overall chemistry between players and staff.

## Figures and Tables

**Table 1:** Training structure and microcycle organization during the training protocols of Coach 1.

Training Protocols	MD + 1 (Starters)	MD + 1 (Non-Starters)	MD − 5	MD − 4	MD − 3	MD − 2	MD − 1
Pre-training	OFF	OFF	N/A	N/A	N/A	N/A	N/A
Warm up	OFF	OFF	Dynamic stretches, rondo, passing drill	Dynamic stretches, rondo, passing drill	Dynamic stretches, rondo, passing drill	Dynamic stretches, rondo	Dynamic stretches, rondo, passing drill, reaction drill
Main body	OFF	OFF	Possession game, SSG	Possession game, SSG	Possession game, tactical game, SSG	Fun games, set pieces	SSG, Tactical play, crossing, finishing, set pieces
Post-training	OFF	OFF	2 laps at a very low pace around the pitch	2 laps at a very low pace around the pitch	2 laps at a very low pace around the pitch	2 laps at a very low pace around the pitch	2 laps at a very low pace around the pitch

**Table 2:** Training structure and microcycle organization during the training protocols of Coach 2.

Training Protocols	MD + 1 (Starters)	MD + 1 (Non-Starters)	MD − 5	MD − 4	MD − 3	MD − 2	MD − 1
Pre-training	N/A	Muscle activation and mobility exercises	OFF	≈30 min in the gym area for resistance-related strength training exercises	≈30 min in the gym area for resistance-related strength training exercises	Muscle activation and mobility exercises	Muscle activation and mobility exercises
Warm up	Muscle activation and mobility exercises	Dynamic stretches, rondos, and passing drills	OFF	Dynamic stretches, speed drill runs (4 × 20 m, 4 × 40 m, and 4 × 50 m), strength-related exercise (runs with resistance bands, weighted squats, deadlift and lunges)	Dynamic stretches, explosive power drills (jumping, acceleration and deceleration exercises), agility runs with hurdles and cones, and short-spaced competitive rondos.	Dynamic stretches, one big rondo, and a short-spaced passing drill	Dynamic stretches, short passing drill, speed reaction drill (10 × 10 m sprints on reaction)
Main body	Resistance-related strength training exercises in the gym area ≈ 50 min		OFF	Possession game, shooting drill	SSG	Possession game, tactical play, set pieces	Rondo, tactical play, set pieces, SSG
Post-training	Static stretches and foam rolling	Static stretches and foam rolling	OFF	Static stretches and foam rolling	Static stretches and foam rolling	Static stretches and foam rolling	Static stretches and foam rolling

**Table 3:** Training structure and microcycle organization during the training protocols of Coach 3.

Training Protocols	MD + 1 (Starters)	MD + 1 (Non-Starters)	MD − 5	MD − 4	MD − 3	MD − 2	MD − 1
Pre-training	OFF	OFF	10 min presentations on the content for the training design	10 min presentations on the content for the training design	10 min presentations on the content for the training design	10 min presentations on the content for the training design	10 min presentations on the content for the training design
Warm up	OFF	OFF	Dynamic stretches, passing drill, small rondos	Dynamic stretches, small rondos	Dynamic stretches, passing drill with scoring	Dynamic stretches, fun games,	Dynamic stretches, one big rondo, speed and reaction drill (5 short accelerations)
Main body	OFF	OFF	LSG, set pieces, crossing and finishing	LSG	LSG with tactical play, crossing and finishing	LSG with tactical play	LSG with tactical play, transition drill, crossing and finishing
Post-training	OFF	OFF	Static stretching	≈10 min provided for additional shooting (for forward positions), and crosses and long passes from backfield players.	≈10 min provided for additional shooting (for forward positions), and crosses and long passes from back-field players.	Static stretching	≈10 min provided for additional shooting (for forward positions), and crosses and long passes from back-field players.

**Table 4:** Descriptive characteristics (Mean ± SD) for the three teams, each under a different manager.

Roster Under Each Manager	Age (years)	Height (cm)	Weight (kg)	Body Fat (%)	VO2max (mL.·kg^−1^·m^−1^)
Coach 1	26.3	180.5	79.9	12.4	57.1
Coach 2	26.5	180.8	80.1	11.7	57.7
Coach 3	27.2	181.3	78.7	10.2	56.8
Season’s mean	26.7 ± 0.47	180.9 ± 0.40	79.6 ± 0.76	11.4 ± 1.12	57.2 ± 0.46

VO2max = maximal aerobic capacity.

## Data Availability

The data that support the findings of this study are available from the corresponding author upon reasonable request. The data are not publicly available due to privacy and ethical restrictions.
